# Involvement of adiponectin in age-related increases in tear production in mice

**DOI:** 10.18632/aging.102322

**Published:** 2019-10-08

**Authors:** Yosuke Shikama, Mie Kurosawa, Masae Furukawa, Naozumi Ishimaru, Kenji Matsushita

**Affiliations:** 1 Department of Oral Disease Research, National Center for Geriatrics and Gerontology, Obu 474-8511, Japan; 2 Department of Oral Molecular Pathology, Tokushima University Graduate School of Biomedical Sciences, Tokushima 770-8504, Japan

**Keywords:** dry eye, adiponectin, peroxisome proliferator-activated receptor gamma, senescence-associated T cells, aging

## Abstract

Common age-related changes in the human eye contribute to the development of dry eye, including decreases in aqueous tear production. Although the infiltration of lymphocytes into the lacrimal glands occurs with age, age-related increases in tear production have also been observed in mice; however, the mechanisms underlying this increase remain unclear. We herein demonstrated that increases in tear production were not dependent on body weight gain or systemic conditions, such as insulin resistance, using aged mice and high-fat diet-fed mice. The results obtained also showed that senescence-associated T (SA-T) cells accumulated in the lacrimal glands of aged mice, particularly females. Expression levels of the nuclear transcription factor peroxisome proliferator-activated receptor-γ (PPARγ) in whole lacrimal glands and epithelial cells isolated from lacrimal glands were significantly higher in aged mice than in young mice. The expression levels of adiponectin and one of its receptors, AdipoR2, also increased in the lacrimal glands of aged mice, but not in those of high-fat diet-fed mice. Collectively, the present results indicate that PPARγ and adiponectin-mediated signaling contribute to age-related increases in tear production in mice and have potential as therapeutic targets for the treatment of dry eye in humans.

## INTRODUCTION

Epidemiological studies have shown that the prevalence of dry eye increases every five years after the age of 50 years, with a higher prevalence being reported in women than in men [1–3]. Age and female sex have been identified as the greatest risk factors for dry eye, and this is supported by the clinical findings of decreased tear production in women through the 6^th^ decade of life [4, 5]. The lacrimal glands are significantly affected by aging. Various histopathological changes, which cause tear dysfunction, have been reported in the lacrimal glands of humans, such as the infiltration of lymphocytes [6, 7]. However, a previous study reported that despite the age-related infiltration of lymphocytes into the lacrimal glands of aged mice, the volume of tears produced was larger in aged mice than in young mice [8]. The mechanisms underlying these increases in tear production in aged mice have not yet been elucidated.

Metabolic disorders, such as diabetes, affect tear production and are associated with dry eye [9, 10]. Regarding the mechanisms responsible for type 2 diabetes, aging is known to induce insulin resistance [11], which is attributed to decreased plasma adiponectin levels in humans [12]. Adiponectin is a 30-kDa multimeric protein that is mainly secreted by white adipose tissue, and has insulin-sensitizing [13], anti-atherogenic, and anti-inflammatory properties [14, 15]. Moreover, adiponectin is secreted from adipocytes into the bloodstream as three oligomeric complexes: a trimer, hexamer, and high-molecular-weight multimer comprising at least 18 monomers [16, 17]. Assembly into the high-molecular-weight form is essential for the function of adiponectin [18]. Globular adiponectin, the globular C1q domain of adiponectin generated from the full-length protein by proteolysis, is also biologically active [19]. AdipoR1 and AdipoR2, two structurally related seven-transmembrane receptors, function as adiponectin receptors. AdipoR1 exhibits high affinity for globular adiponectin and low affinity for full-length adiponectin. On the other hand, AdipoR2 mainly recognizes full-length adiponectin [20]. The nuclear transcription factor peroxisome proliferator-activated receptor-γ (PPARγ) is a major regulator of adipocyte function and controls the secretion of adipokines, particularly adiponectin [21–23]. Previous studies reported that a PPARγ ligand and adiponectin exerted therapeutic effects on tear production [24, 25].

In the present study, we examined the involvement of insulin resistance in stimulated tear secretion in aged [26] and high-fat diet-fed [27] mice. Although the volume of tears secreted increased with age, it slightly or significantly decreased in high-fat diet-fed mice in a gender-dependent manner. Neither high-fat diet feeding nor aging exerted significant effects on the mRNA expression levels of muscarinic acetylcholine receptor M3 (M3R), a selective receptor of pilocarpine, in the lacrimal glands. Moreover, we confirmed that not only lymphocytes, as previously reported [8], but also senescence-associated T (SA-T) cells, which mainly accumulate in lymphoid tissues with age [28], also accumulated in the lacrimal glands of aged mice. PPARγ mRNA expression levels significantly increased in lacrimal glands as well as primary epithelial cells isolated from the lacrimal glands of aged mice. Adiponectin mRNA levels significantly increased in the white adipose tissue of aged mice. The present results also revealed that adiponectin and adipoR2 mRNA expression levels significantly increased in the lacrimal glands of aged mice, but not in those of high-fat diet-fed mice. These results indicate that PPARγ and adiponectin-mediated signaling is involved in age-related increases in tear production in mice.

## RESULTS

### Increases in pilocarpine-stimulated tear secretion in aged mice, but not in high-fat diet-fed mice

**Figure 1:**
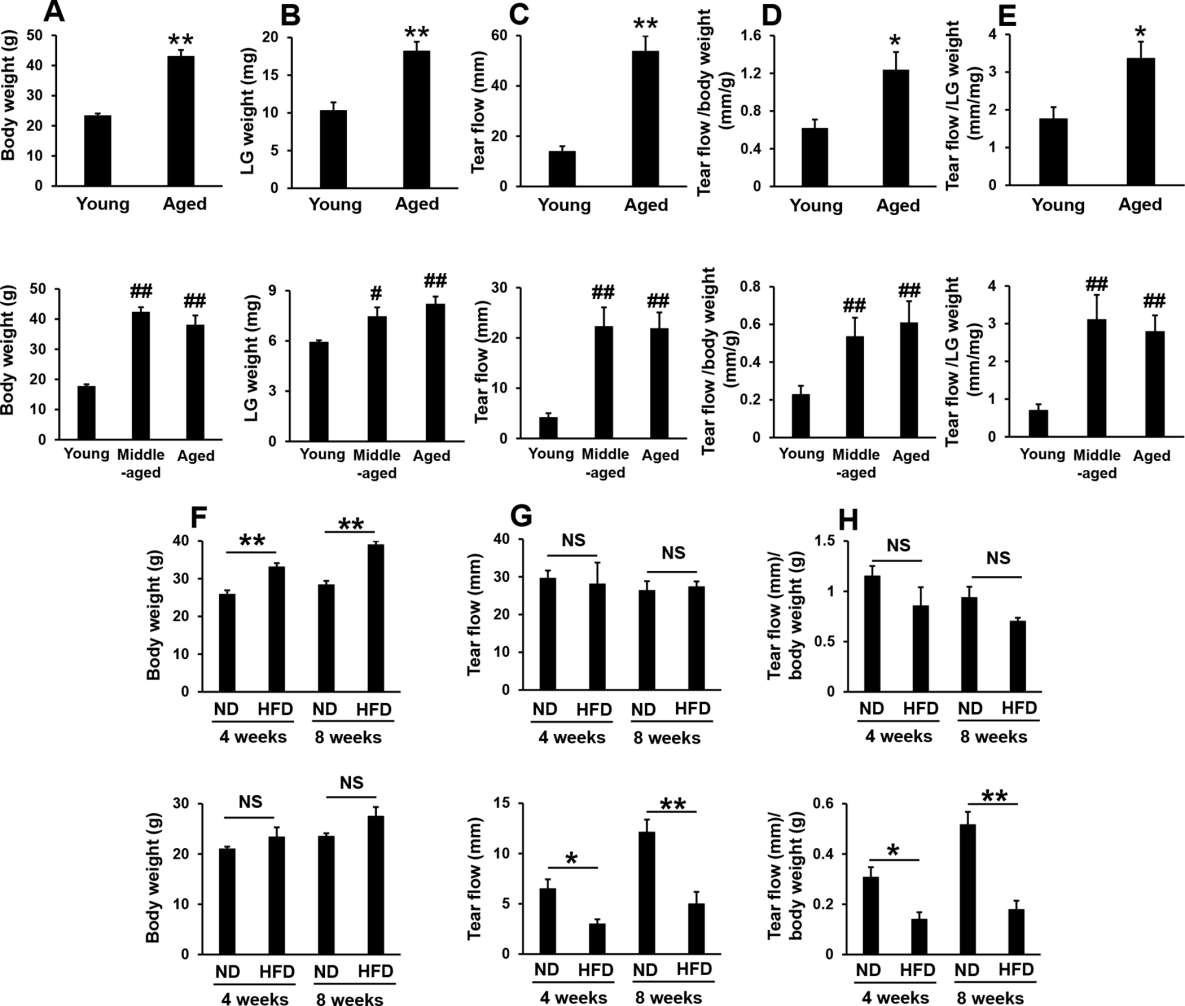
**Pilocarpine-stimulated tear secretion increased in aged mice, but not in high-fat diet-fed mice.** (**A** and **B**) Body weights and the weights of the lacrimal glands (LG) in young, middle-aged, and aged mice. Upper and lower graphs show male (N=5) and female mice (N=4-6), respectively. (**C**–**E**) Absolute volume of tear flow ©, adjusted by body weight (**D**) or LG weight (**E**). Upper and lower graphs show male (N=6) and female mice (N=4-6), respectively. (**F**) Body weight in normal diet (ND)- or high-fat diet (HFD)-fed mice for the indicated period (N=4). Upper and lower graphs show male and female mice, respectively. (**G** and **H**) Absolute volume of tear flow (**G**) adjusted by body weight (**H**) in ND or HFD-fed mice for the indicated period (N=4). Upper and lower graphs show male and female mice, respectively. Values are presented as means ± SEM. *p<0.05 and **p<0.01 (an unpaired Student’s *t*-test). #p<0.05 and ##p<0.01 versus young mice (Dunnett’s multiple comparison test).

Body weight and the weight of the lacrimal glands were significantly higher in aged than in young male and female mice (Figure 1A, 1B). The volume of tears secreted also significantly increased with aging (Figure 1C), even when volumes were adjusted for body weight (Figure 1D) or the weight of the lacrimal glands (Figure 1E). However, although body weight significantly or slightly increased in high-fat diet-fed male or female mice, respectively (Figure 1F), the volume of tears secreted significantly decreased in high-fat diet-fed female, but not male mice (Figure 1G, 1H). These results suggest that the volume of stimulated tear secretion was not dependent on body weight or systemic conditions, such as insulin resistance.

### Effects of aging or high-fat diet feeding on M3R mRNA expression in murine lacrimal glands

**Figure 2:**
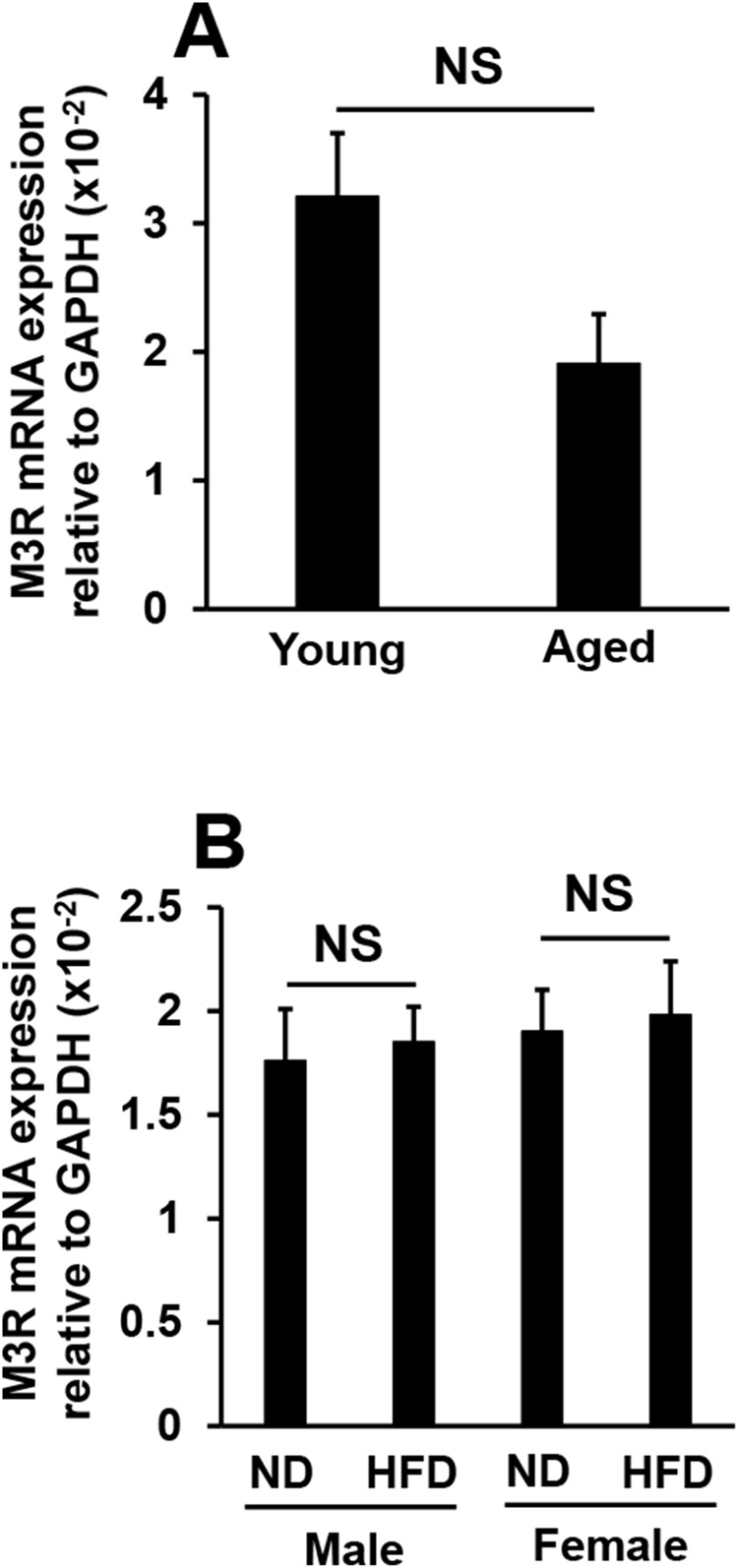
Effects of aging or high-fat diet feeding on M3R mRNA expression in lacrimal glands. M3R mRNA expression levels in young and aged mice (N=7-8) (**A**), and in mice fed a normal diet (ND) or high-fat diet (HFD) for 8 weeks (N=4-5) (**B**). Values are presented as means ± SEM. NS, not significant (an unpaired Student’s *t*-test).

M3R is strongly expressed in the lacrimal glands and is involved in the secretion of tears. Therefore, we examined M3R expression levels in the lacrimal glands of aged and high-fat diet-fed mice. No significant differences were observed in M3R mRNA expression levels between young and aged mice; expression levels were slightly lower in aged mice than in young mice (Figure 2A). On the other hand, no significant differences were noted in M3R mRNA expression levels between high-fat diet-fed mice and normal diet-fed mice independent of sex (Figure 2B). These results indicate that the increases and decreases observed in the volume of tears secreted in aged mice and high-fat diet-fed mice, respectively, were not dependent on M3R expression levels in the lacrimal glands.

### SA-T cell accumulation in lacrimal glands of aged mice

**Figure 3:**
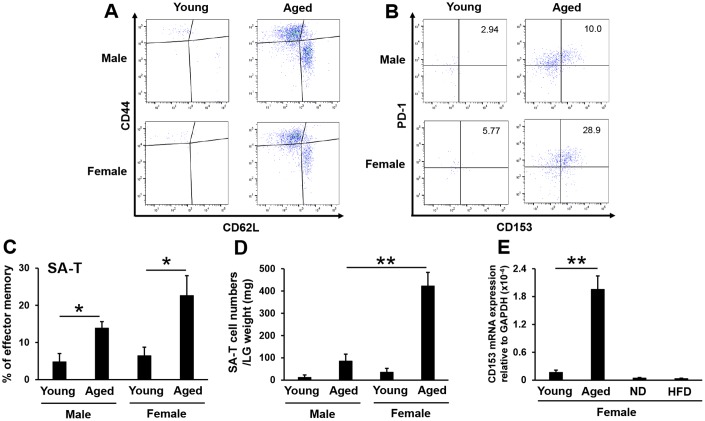
**SA-T cells accumulate in lacrimal glands of aged mice, particularly in female mice.** (**A** and **B**) Naïve (CD44^lo^CD62L^hi^) and effector memory (CD44^hi^CD62L^lo^) expression in CD4^+^ T cells (**A**), or PD-1^+^CD153^+^ expression in effector memory CD4^+^ T cells (**B**) in the lacrimal glands of young and aged mice. Results are representative of those from each group of mice. (**C** and **D**) Proportions (N=5) (**C**) and numbers adjusted by the lacrimal gland (LG) weight (N=4) (**D**) of PD-1^+^CD153^+^ cells gated on effector memory CD4^+^ T cells in the LG of young and aged mice. (**E**) CD153 mRNA expression levels in the LG of young and aged female mice, and of female mice fed a normal diet (ND) or high-fat diet (HFD) for 8 weeks (N=4-5). Values are presented as means ± SEM. *p<0.05 and **p<0.01 (an unpaired Student’s *t*-test).

The immune system undergoes aging, a typical feature of which is a chronic, low-grade inflammatory status called inflammaging [29], which is characterized by a general increase in the production of pro-inflammatory cytokines. Significant changes also occur in overall T-cell populations with age. In CD4^+^ T cells, the populations of naïve (CD44^lo^CD62L^hi^) T cells markedly decline in ontogeny, with age-dependent increases occurring in effector memory T cells (CD44^hi^CD62L^lo^) [30, 31]. Among effector memory CD4^+^ T cells, a unique population, SA-T cells, which express programmed cell death 1 (PD-1) and CD153, increase in lymphoid tissues with aging [28]. Moreover, Shirakawa et al. reported that SA-T cells also accumulated in the visceral adipose tissue of high-fat diet-fed mice, which induced chronic inflammation in visceral adipose tissue and insulin resistance [32]. We confirmed that effector memory CD4^+^ T cells markedly increased in the lacrimal glands of male and female aged mice (Figure 3A). The proportion of SA-T cells also significantly increased in male and female aged mice (Figure 3B, 3C), and the number of SA-T cells was approximately four-fold higher in female aged mice than in male aged mice (Figure 3D). Moreover, we confirmed that SA-T cells did not accumulate in the lacrimal glands of high-fat diet-fed mice based on CD153 mRNA expression levels (Figure 3E). These results demonstrated that SA-T cells accumulated in the lacrimal glands of aged, but not high-fat diet-fed mice.

### PPARγ expression in lacrimal glands and adiponectin mRNA expression in the white adipose tissue of aged mice

**Figure 4:**
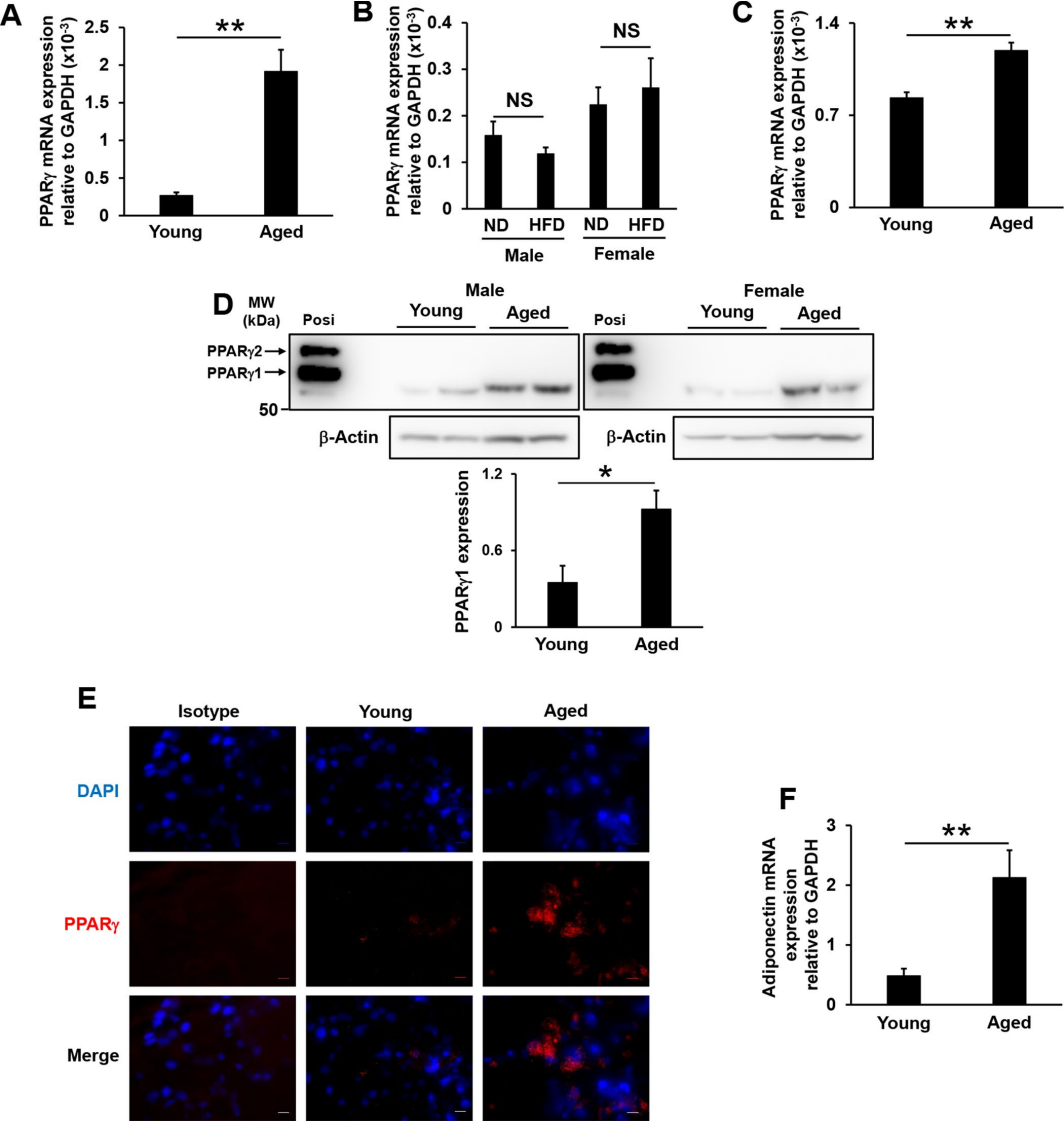
**PPARγ expression in lacrimal glands and adiponectin mRNA expression in the white adipose tissue of aged and high-fat diet-fed mice.** (**A** and **B**) PPARγ mRNA expression levels in the lacrimal glands of young and aged mice (N=7-8) (**A**), or of mice fed a normal diet (ND) or high-fat diet (HFD) for 8 weeks (N=4-5) (**B**). (**C**) PPARγ mRNA expression levels in the epithelial cells of the lacrimal glands of young and aged mice (N=4). (**D**) Detection of the PPARγ protein by Western blotting. Lysates prepared from the lacrimal glands of young and aged mice were immunoblotted with anti-AdipoR2 and anti-β-Actin antibodies. Left and right images show male (N=2) and female mice (N=2), respectively. The positive control (Posi) is a lysate prepared from the subcutaneous fat of young mice. The bar graph shows integrated signal intensities in AdipoR2 normalized to that of β-Actin (N=4). (**E**) PPARγ expression in the acinar cells of the lacrimal glands of young and aged mice as detected by immunofluorescence. Nuclei were stained with DAPI. Bars = 10 μm. (**F**) Adiponectin mRNA expression levels in the mesenteric white adipose tissues of young and aged mice (N=4-5). Values are presented as means ± SEM. NS, not significant. **p<0.01 (an unpaired Student’s *t*-test).

PPARγ expression levels in the white adipose tissue of humans and a rodent model were shown to significantly decrease with aging [33]. However, in the present study, PPARγ mRNA expression levels in the lacrimal glands significantly increased in aged mice (Figure 4A), but not in high-fat diet-fed mice (Figure 4B). By isolating primary epithelial cells from the lacrimal glands, we also confirmed that these expression levels significantly increased in epithelial cells isolated from aged mice (Figure 4C). PPARγ exists as two isoforms, PPARγ1 and PPARγ2, with the latter containing an additional 30 amino acids at its N terminus. PPARγ1 is expressed in many tissues, while the expression of PPARγ2 is restricted to adipose tissue under physiological conditions [34]. We found that PPARγ1 protein levels were significantly increased in the lacrimal glands of aged mice (Figure 4D), and PPARγ was detected in both the cytoplasm and nuclei, with higher expression levels in the cytosol of acinar cells (Figure 4E). A previous study reported that adiponectin mRNA levels in adipose tissue significantly decreased in high-fat diet-fed mice and obese volunteers [35]. However, although body weight and the volume of white adipose tissue in mice increased with age, adiponectin mRNA levels significantly increased in the white adipose tissue of aged mice (Figure 4F). Since a PPARγ ligand and adiponectin have been shown to enhance tear secretion [24, 25], the present results indicate that increases in PPARγ expression levels in the lacrimal glands and in adiponectin expression levels in white adipose tissue are involved in age-related increases in tear secretion in mice.

### Effects of aging and high-fat diet feeding on adiponectin, adipoR1, and adipoR2 expression levels in lacrimal glands

**Figure 5:**
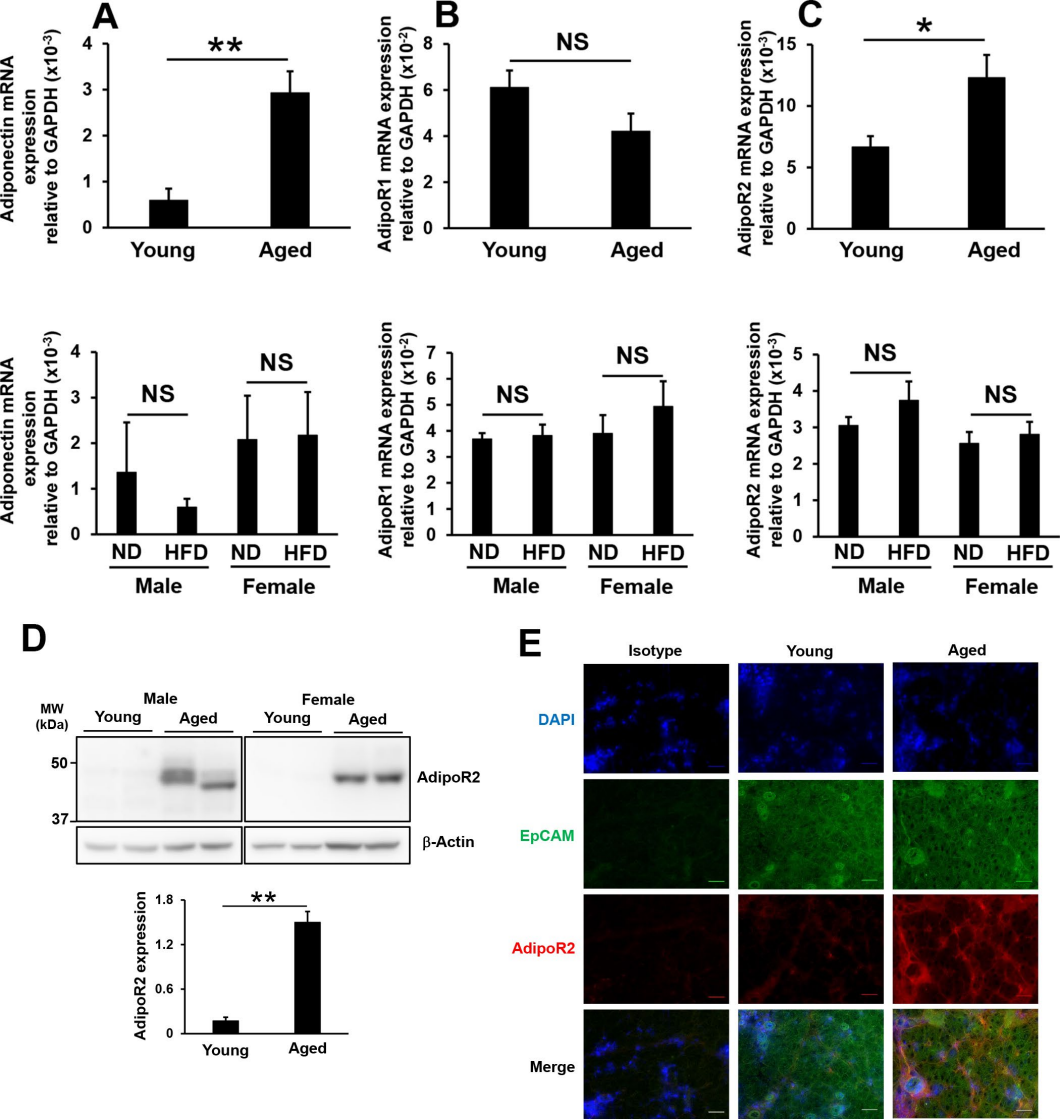
**Influence of aging or high-fat diet feeding on adiponectin, adipoR1, and adipoR2 expression in lacrimal glands.** Adiponectin (**A**), adipoR1 (**B**), and adipoR2 (**C**) mRNA expression levels in lacrimal glands. Upper and lower graphs show results in young and aged mice (N=7-8), and in mice fed a normal diet (ND) or high-fat diet (HFD) for 8 weeks (N=4-5), respectively. (**D**) Detection of the AdipoR2 protein by Western blotting. Lysates prepared from the lacrimal glands of young and aged mice were immunoblotted with anti-AdipoR2 and anti-β-Actin antibodies. Left and right images show male (N=2) and female mice (N=2), respectively. The bar graph shows integrated signal intensities in AdipoR2 normalized to that of β-Actin (N=4). (**E**) AdipoR2 expression in the lacrimal glands of young and aged mice as detected by immunofluorescence. Nuclei were stained with DAPI. Bars = 40 μm. Values are presented as means ± SEM. NS, not significant. *p<0.05 and **p<0.01 (an unpaired Student’s *t*-test).

AdipoR1 and AdipoR2 are expressed in murine lacrimal glands [25], and the administration of 5-aminoimidazole-4-carboxamide ribonucleoside (AICAR), which is an activator of adenosine monophosphate-activated protein kinase (AMPK), similar to adiponectin, induced tear secretion in mice [36]. Therefore, we investigated adiponectin, adipoR1, and adipoR2 expression levels in the lacrimal glands of aged and high-fat diet-fed mice. The mRNA expression levels of adiponectin significantly increased in aged mice, but not in high-fat diet-fed male or female mice (Figure 5A). No significant changes were observed in adipoR1 mRNA expression levels in aged or high-fat diet-fed mice (Figure 5B). The mRNA expression levels of adipoR2, which is mainly activated by full-length adiponectin and, in turn, activates AMPK, were significantly increased in aged mice, but not in high-fat diet-fed mice (Figure 5C). In a Western blotting analysis, we confirmed that AdipoR2 protein levels were markedly increased in the lacrimal glands of aged mice (Figure 5D). Moreover, EpCAM-positive cells expressed AdipoR2 in the lacrimal glands of aged mice, particularly in duct cells and the basal membrane of acinar cells (Figure 5E). Based on the results shown in Figure 4, age-related increases in tear secretion may be due to elevated expression levels of PPARγ, adiponectin, and adipoR2 in the lacrimal glands as well as increased adiponectin expression levels in white adipose tissue.

## DISCUSSION

Although age and female sex are the greatest risk factors for dry eye in humans, we and other researchers [8, 37] showed that aqueous and pilocarpine-stimulated aqueous tear production both paradoxically increased with age in mice. The plasma levels of high-molecular-weight adiponectin, which may represent the most biologically active form of adiponectin [38, 39], were previously shown to decrease with age in women only [40], whereas high-molecular-weight adiponectin levels in plasma [41] and adiponectin mRNA expression in white adipose tissue (Figure 4F) both increased with age in mice. These discrepancies between human and murine adiponectin levels may result in paradoxical tear secretion with age. We also confirmed that the volume of tears secreted significantly decreased in high-fat diet-fed female, but not male mice (Figure 1G, H). Comb et al*.* reported that increases in plasma adiponectin levels during sexual maturation were 2.5-fold larger in female mice than in male mice [42]. In the present study, we fed mice a normal or high-fat diet from 4 weeks of age for 4 or 8 weeks, and sexual maturation occurred during this period. Since adiponectin mRNA levels in adipose tissue significantly decreased in high-fat diet-fed mice [35], plasma adiponectin levels may markedly decrease if feeding of the high-fat diet starts before or during sexual maturation in female mice, resulting in reductions in tear secretion presumably via an adiponectin-mediated pathway.

In addition to previous findings showing mild or moderate lymphocytic infiltration in the lacrimal glands of aged mice [8, 43], we also noted an increase in the accumulation of SA-T cells in the lacrimal glands with age (Figure 3). The elevated tear volume in aged mice suggests that this level of inflammation is not sufficient to decrease secretory function. SA-T cells express PD-1, which a negative costimulatory receptor for T-cell receptor (TCR) signaling [44], and CD153, which is a tumor necrosis factor (TNF) superfamily protein [45]. SA-T cells show compromised proliferation and express large amounts of proinflammatory cytokines, such as osteopontin [28]. We demonstrated that the number of SA-T cells was approximately four-fold higher in female aged mice than in male aged mice (Figure 3D). However, McClellan et al*.* reported that the CD4^+^ T-cell population was larger in 24-month-old male mice than in female mice of the same age [8]. In humans [46] and mice [47], senescent CD4^+^ T cells have been shown to play a role in the pathogenesis of rheumatoid arthritis and systemic lupus erythematosus, which are female-dominant systemic autoimmune diseases. These findings indicate that the systemic conditions of females may be more inducible to senescent CD4^+^ T cells than those of males. In the present study, we evaluated CD153 mRNA levels as a marker for SA-T cells (Figure 3E). In mice, CD153 has been detected on activated CD4^+^ T cells and dendritic cells [48]. Infiltrated CD4^+^ T cells in the lacrimal glands of aged mice were composed of the naïve or memory phenotype (Figure 3A). We confirmed that CD8^+^ and naïve CD4^+^ T cells did not express CD153 using a flow cytometric analysis (Supplementary Figure 1). Regarding DC, McClellan et al*.* previously reported that CD11b^+^ major histocompatibility complex (MHC) II^+^ cells and CD11c^+^ MHC II^+^ cells decreased [8], and we also confirmed that CD11b^+^ CD11c^+^ cells significantly decreased in the lacrimal glands of aged mice (Supplementary Figure 2). Based on these results, it is reasonable to consider CD153 mRNA expression levels as a SA-T cell marker in the lacrimal glands using a real-time PCR analysis.

In recent years, evidence has been accumulating to support the potential benefits of PPARγ, which attenuates or prevents eye diseases. Chen et al. [24] reported that the expression of PPARγ was down-regulated in the conjunctiva of mice with dry eye, and they also found that pioglitazone, a synthetic PPARγ ligand, exerted therapeutic effects to increase tear fluid production and enhance tear film stability. Moreover, the localization of PPARγ was shown to be involved in age-related changes in the meibomian glands, resulting in meibomian gland dysfunction [49, 50]. We and other researchers reported the accumulation of lymphocytes in the lacrimal glands of aged mice. PPARγ is expressed on some immune cells, such as macrophages, B lymphocytes, and T lymphocytes [51–53]. By isolating epithelial cells from the lacrimal glands, we revealed that PPARγ mRNA expression levels were up-regulated in aged mice (Figure 4C). Further experiments are needed to elucidate the underlying molecular mechanisms and demonstrate the efficacy of PPARγ ligands for the treatment of dry eye diseases.

IL-6 and TNF-α contribute to the senescence-associated secretory phenotype (SASP) [54], and their concentrations were found to be increased in the serum of both high-fat diet fed [55] and aged [56] mice. Moreover, the levels of these cytokines in tear fluid were shown to be higher in dry eye patients than in control subjects [57–59]. Leptin is mainly produced by adipocytes and is regarded as a proinflammatory adipokine because it appears to contribute to the so-called low-grade inflammatory state in overweight and obese individuals [60]. Leptin concentrations were previously shown to be increased in the serum of high-fat diet fed and aged mice [61]. The present results showed that the volume of tears was increased in aged (Figure 1C–1E), but not high-fat fed (Figure 1G and 1H) mice even when IL-6, TNF-α, and leptin concentrations were elevated in the serum of these mice. Furthermore, no significant differences were observed in IL-6 mRNA levels (we were unable to detect leptin even when the threshold cycle value was more than 40 cycles) in the epithelial cells of lacrimal glands between young and aged mice, whereas IL-6 and leptin mRNA levels were significantly higher in the lacrimal glands of aged mice (Supplementary Figure 3), suggesting that the source of IL-6 and leptin mRNA is not epithelial cells (possibly infiltrated lymphocytes).

In conclusion, the present results demonstrated the accumulation of SA-T cells in aged mice, which occurred to a greater extent in female than in male mice. Furthermore, increased tear secretion in aged mice appeared to be mediated by PPARγ and adiponectin-mediated signaling. These results may explain the discrepancy in the volume of tears secreted with age between humans and mice.

## MATERIALS AND METHODS

### Animals

All animal experiments were approved by and conducted in accordance with guidelines established by the National Center for Geriatrics and Gerontology Animal Ethics Committee. Young adult C57BL/6N mice (age: 8-10 weeks), middle-aged adult C57BL/6N mice (age: 12 months), and aged adult C57BL/6N mice (age: 22-25 months) were obtained from Japan SLC Inc. (young and middle-aged) or the Experimental Animal Facility at the National Center for Geriatrics and Gerontology (aged: Obu, Japan). In high-fat diet experiments, mice were fed a normal diet (CE-2) or high-fat diet (HFD-32) from 4 weeks of age, as described in the Figure legends. These diets were purchased from CLEA Japan, Inc. Mice were housed in specific pathogen-free conditions under a 12-h light-dark photocycle and had *ad libitum* access to water and the diet. The temperature in the room was maintained at 23 ± 2°C and 50 ± 10% humidity.

### Measurement of tear flow rates

The phenol red thread test was used to measure the stimulated flow rates of tears as previously described [62]. Briefly, individual mice were weighed, anesthetized, and intraperitoneally injected with 1 mg/kg of pilocarpine (Kanto Chemical Co., Inc.). Five min later, tear volumes were measured using a phenol red thread (ZONE-QUICK, Ayumi Pharmaceutical Corporation), which was placed on the medial canthus. The total volume of tears was normalized by body weight or the weight of the lacrimal glands, as described in the Figure legends.

### Quantitative real-time PCR analysis

Total RNA was extracted from cells and tissues using an RNeasy mini kit or RNeasy Lipid Tissue Mini Kit (Qiagen), respectively, according to the manufacturer’s instructions. Total RNA concentrations were measured using a Nanodrop spectrophotometer (Thermo Fisher Scientific), and cDNA was synthesized with the PrimeScript RT Master Mix (Takara Bio Inc.). PCR was performed on a LightCycler 96 system using FastStart Essential DNA Green Master (Roche Applied Science). The following primers were used for the amplification of specific genes: adiponectin, 5′- CAGGCATCCCAGGACATCC-3′ (sense) and 5′- CCAAGAAGACCTGCATCTCCTTT-3′ (antisense); AdipoR1, 5′- AGTTCATGTATAAGGTCTGGGAGG-3′ (sense) and 5′-CACATCTACGGGATGACTCTCCA-3′ (antisense); AdipoR2, 5′-TTCCTATTATGAAAATAGCCCGGA-3′ (sense) and 5′-CATGATGGGAATGTAGGAGC-3′ (antisense); GAPDH, 5′-GCCTTCCGTGTTCCTACCC-3′ (sense) and 5′-TGAAGTCGCAGGAGACAACC-3′ (antisense); interleukin (IL)-6, 5′-CCACTTCACAAGTCGGAGGCTTA-3′ (sense) and 5′-GCAAGTGCATCATCGTTGTTCATAC-3′ (antisense); leptin, 5′-CAAGCAGTGCCTATCCAGA-3’ (sense) and 5′-AAGCCCAGGAATGAAGTCCA-3′ (antisense); M3R, 5′-AGAGCTGGAAGCCCAGTGC-3′ (sense) and 5′-GTAGCTTGGTAGAGTTGAGGATGG-3′ (antisense); PPARγ, 5′-TTTTCAAGGGTGCCAGTTTC-3′ (sense) and 5′-AATCCTTGGCCCTCTGAGAT-3′ (antisense). The murine CD153 primer (Mm_Tnfsf8_1_SG QuantiTect Primer Assay) was obtained from Qiagen. The relative mRNA expression of each transcript was normalized against GAPDH mRNA.

### Flow cytometric analysis

Immune cells from lacrimal glands were stained using PE-Cy7-conjugated anti-mouse CD4 mAb (GK1.5; BioLegend), PE-Cy5-conjugated anti-mouse CD8a mAb (53-6.7; BioLegend), PE-Cy5-conjugated anti-mouse/human CD11b mAb (M1/70; BioLegend), allophycocyanin (APC)-Cy7-conjugated anti-mouse CD11c mAb (N418; BioLegend), PE-Cy7-conjugated anti-mouse CD45.2 (Ly5.2) mAb (104; BioLegend), APC-conjugated anti-mouse CD44 mAb (IM7; BioLegend), APC-Cy7-conjugated anti-mouse CD62L mAb (Mel-14; BioLegend), PE-conjugated anti-mouse CD153 mAb (RM153; BioLegend), fluorescein isothiocyanate (FITC)–conjugated anti-mouse CD279 (PD-1) mAb (29F; BioLegend), and FITC–conjugated anti-mouse CD326 (epithelial cell adhesion molecule; EpCAM) mAb (G8.8; BioLegend). A Canto II flow cytometer (BD Biosciences) was used to identify cell populations according to surface expression profiles. Flow cytometric data were analyzed using FlowJo software (BD Biosciences). The representative gating strategy for SA-T cells is shown in Supplementary Figure 4.

### Isolation of epithelial cells from lacrimal glands using MACS

Lacrimal glands were minced in Dulbecco’s modified Eagle medium (DMEM) containing 10% fetal bovine serum (FBS) and 100 U/ml Penicillin Streptomycin (Pen Strep, Thermo Fisher Scientific), and digested at 37°C for 40 min with 1 mg/ml collagenase type I (Wako), 1 mg/ml hyaluronidase type I (Sigma), 0.01 mg/ml DNAse I (Roche), and 100 U/ml Pen Strep in DMEM. After being digested, they were filtered through a 70-μm nylon mesh, centrifuged, and rinsed twice with DMEM containing 10% FBS. Epithelial cells from the cell suspension were collected by positive selection using Miltenyi mouse CD326 (EpCAM) MicroBeads. We confirmed that purity was more than 80% (Supplementary Figure 5).

### Western blot analysis

Lacrimal glands or subcutaneous fat was lysed in RIPA buffer supplemented with a protease and phosphatase inhibitor cocktail (Thermo Fisher Scientific) using a disposable homogenizer (BioMasher II; Nippi Inc.). Lysates were centrifuged at 12,000×*g* at 4°C for 10 min, and supernatants were collected. Protein concentrations were assessed using the BCA protein assay kit (Thermo Fisher Scientific). Protein concentrations were adjusted, and samples were then diluted in 2× Laemmli Sample Buffer. After boiling at 95°C for 5 min, proteins were separated using sodium dodecyl sulfate-polyacrylamide gel electrophoresis and transferred to polyvinylidene difluoride membranes (Bio-Rad Laboratories). Membranes were incubated with antibodies against AdipoR2 (sc-514045, Santa Cruz Biotechnology), β-Actin (3598R-100, BioVision), and PPARγ (2443, Cell Signaling Technology). To detect AdipoR2 and PPARγ, antibodies were diluted with Can Get Signal (Toyobo). Proteins were visualized with Immunostar (Wako) and Amersham Imager 680, and the optical densities of protein bands were measured with Amersham Imager 680 Analysis Software (GE Healthcare). Band intensities were normalized to that of β-Actin.

### Immunofluorescence staining

Frozen sections of lacrimal gland tissue were fixed with methanol/acetone (1:1), blocked using 5% normal goat serum (WAKO)/0.3% Triton×-100 (Sigma) in phosphate-buffered saline (PBS), and stained with FITC anti-mouse CD326 (EpCAM) (118207, BioLegend), AdipoR2 (sc-514045), and PPARγ (2443) antibodies. Alexa Fluor594-conjugated anti-mouse IgG (H+L) (8890, Cell Signaling Technology) and Alexa Fluor 555-conjugated anti-Rabbit IgG (H+L) (A21428, Thermo Fisher Scientific) were used as secondary antibodies. These antibodies were diluted with Can Get Signal immunostain solution (Toyobo). After washing 3 times with PBS, nuclear DNA was stained with ProLong Diamond Antifade Mountant with DAPI (Thermo Fisher Scientific). Sections were observed using a fluorescence microscope (KEYENCE) at a magnification of 400× or 1000×.

### Statistical analysis

The significance of differences was evaluated by an unpaired Student’s *t*-test or Dunnett’s multiple comparison test after an analysis of variance (ANOVA) using GraphPad InStat (version 3.10, GraphPad InStat Software Inc.). Values of *p* < 0.05 were considered to be significant.

## Supplementary Material

Supplementary Figures
