# Supplementary material for: Involvement of adiponectin in age-related increases in tear production in mice
Source: Aging (Albany NY). 2019 Oct 8;11(19):8329–46. doi: 10.18632/aging.102322 (PMC6814586; doi:10.18632/aging.102322)
Supplement: Supplementary Figures [file aging-11-102322-s001.pdf]

SUPPLEMENTARY FIGURES

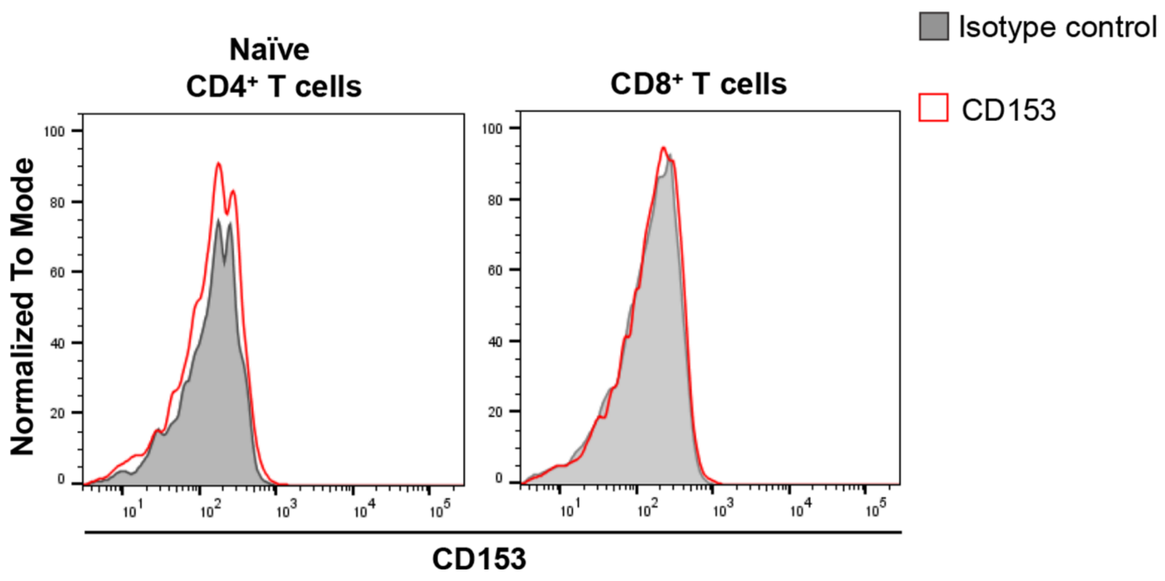

Supplementary Figure 1. CD153 expression in naïve CD4^{+} and CD8^{+} T cells. Flow cytometric analysis of CD153 expression on naïve (CD44^{lo}CD62L^{hi}) CD4^{+} and CD8^{+} T cells in the spleens of aged mice.

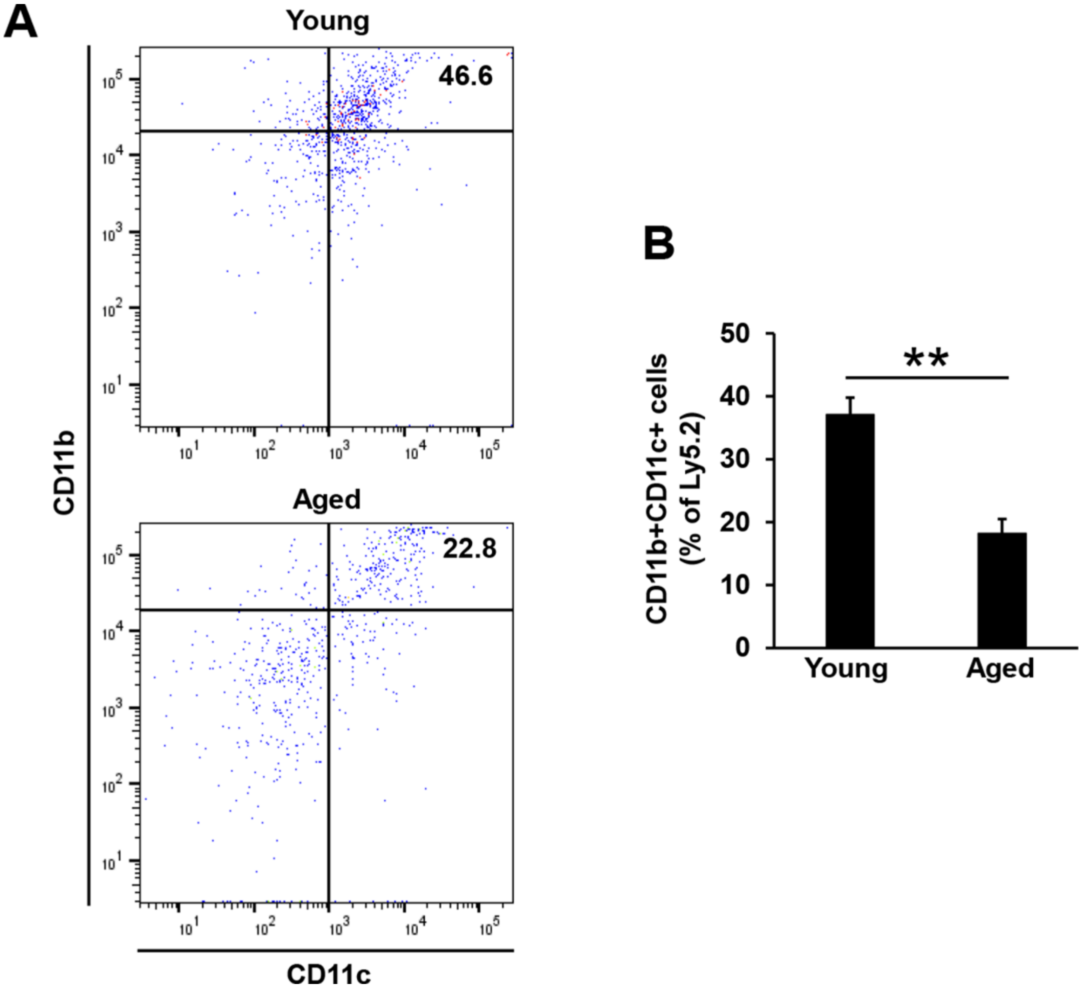

Supplementary Figure 2. CD11b⁺CD11c⁺ cells in lacrimal glands of young and aged mice. Proportion of CD11b⁺CD11c⁺ cells gated on Ly5.2⁺ cells in the lacrimal glands of young and aged mice (Male: N=5-6, Female: N=3-4). Cell debris and lymphocytes were excluded from the analysis based on scatter signals. Values are presented as means ± SEM. **p<0.01 (an unpaired Student's *t*-test).

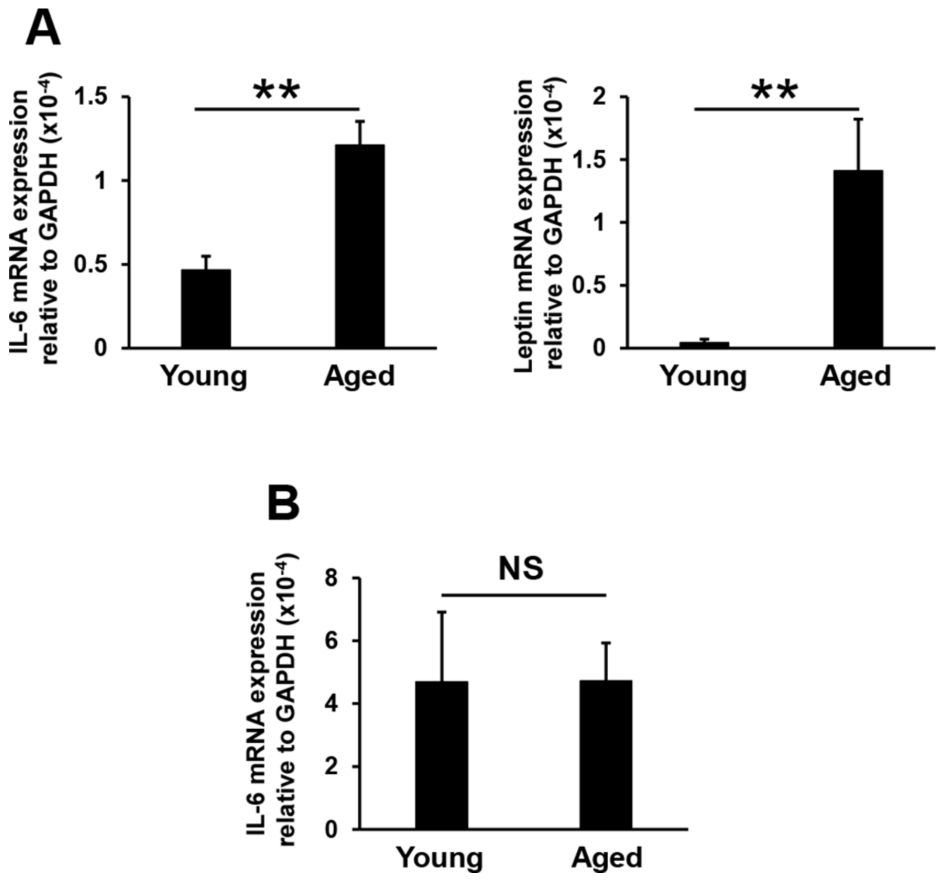

Supplementary Figure 3. IL-6 and leptin mRNA expression in lacrimal glands. (**A**) IL-6 and leptin mRNA expression levels in the lacrimal glands of young and aged mice (N=7-8). (**B**) IL-6 mRNA expression levels in the epithelial cells of the lacrimal glands of young and aged mice (N=4). Values are presented as means ± SEM. NS, not significant. **p<0.01 (an unpaired Student's *t*-test).

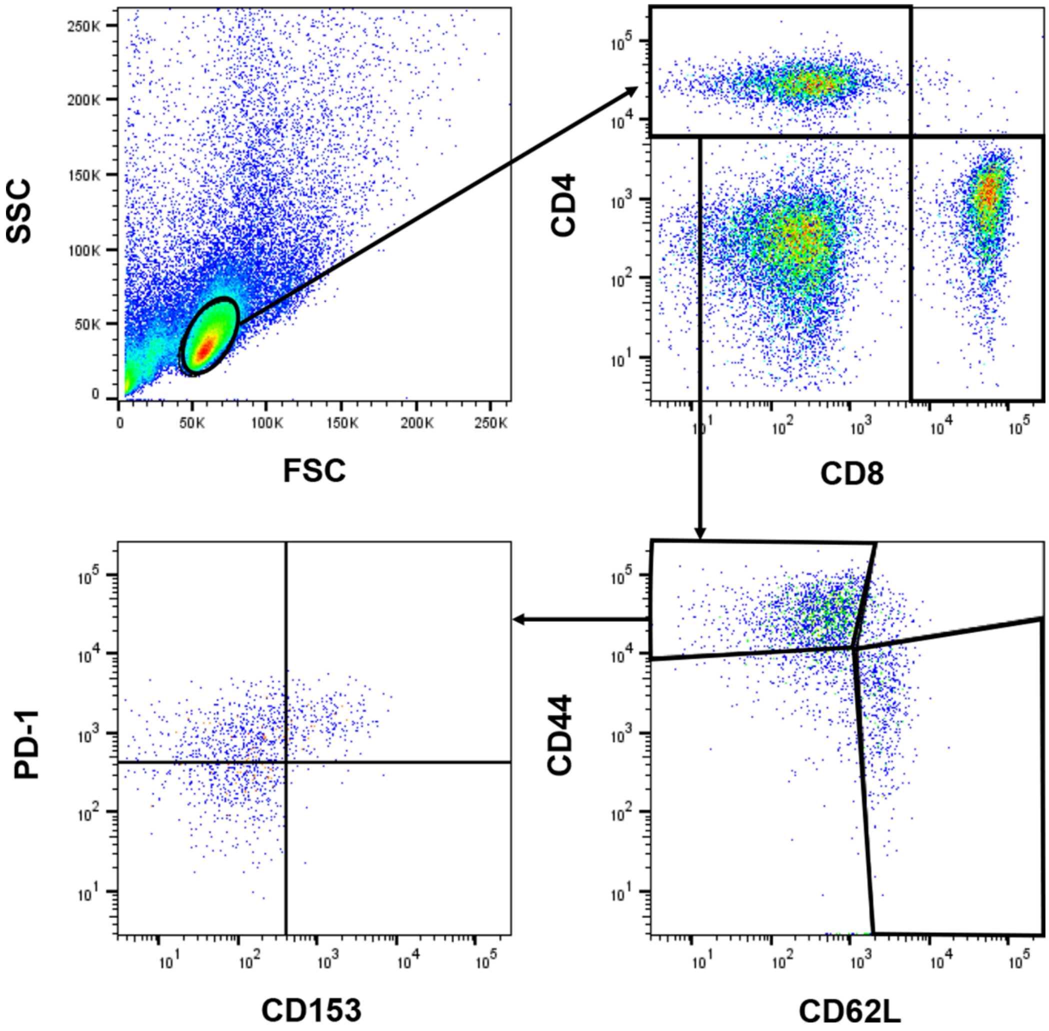

Supplementary Figure 4. Gating strategy for SA-T cells.

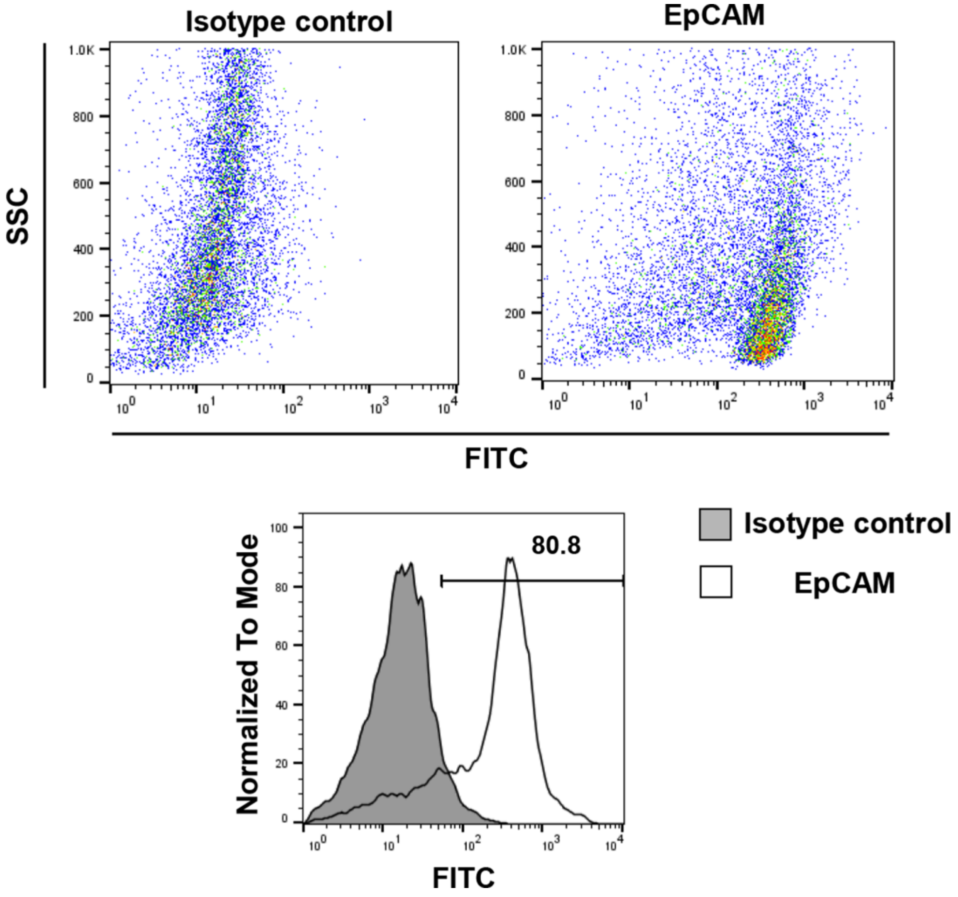

Supplementary Figure 5. Purity of CD326 (EpCAM)-positive cells isolated from lacrimal glands. Epithelial cells were isolated using CD326 (EpCAM) Microbeads from murine lacrimal glands. Cells were stained with an isotype control or FITC-conjugated CD326 (EpCAM) antibody, and analyzed by flow cytometry. Cell debris was excluded from the analysis based on scatter signals.
